# Sperm cryopreservation incidence in men with testicular cancer: towards a stabilization in testicular cancer incidence? Results from the CECOS network

**DOI:** 10.1186/s12610-018-0075-1

**Published:** 2018-08-03

**Authors:** Marie Walschaerts, Louis Bujan, Cécile Chouquet, Valentine Rossi, Jean-Claude Juillard, Patrick Thonneau, Fédération Française des CECOS, Marius Teletin, Aline Papaxanthos-Roche, Florence Brugnon, Marie-Ange Clarotté, Ethel Szerman, Dominique Le Lannou, Celia Ravel, Claire Barthelemy, Fabrice Guerif, Béatrice Delepine, Jean-Luc Bresson, Oxana Blagosklonov, Nathalie Rives, Florence Eustache, Isabelle Berthaut, Rachel Levy, Véronique Drouineaud, Jacques Auger, Vanessa Loup, Catherine Diligent, Isabelle Koscinski, Myriam Daudin, Nathalie Moinard, Bérangère Ducroq, Valérie Mitchell, Sylvie Mirallié, Paul Barriere, Aviva Devaux, Emmanuelle Thibault, Jacqueline Saias-Magnan, Catherine Metzler-Guillemain, Jean-François Guerin, Sylviane Hennebicq

**Affiliations:** 1 Université de Toulouse, UPS, Groupe de Recherche en Fertilité Humaine (EA 3694, Human Fertility Research Group), TSA 70034, 31059 Toulouse Cedex 9, France; 2 CECOS Groupe d’Activité de Médecine de la Reproduction, CHU Toulouse, Toulouse, France; 3 Institut de Mathématiques de Toulouse, Laboratoire de Statistique et Probabilités, CNRS (UMR 5219), Paul Sabatier Université, Toulouse, France; 4 AP-HP CCS SI Patient, Hôpital Rothschild, Paris, France

**Keywords:** Testicular cancer, Sperm cryopreservation, Cancer trends, Statistical models, Cancer du testicule, Autoconservation de sperme, Tendances, Modèles statistiques

## Abstract

**Background:**

Testicular cancer (TC) represents 1% of all new male cancer cases but remains the most frequent cancer in adolescents and young adults in industrialized countries. In this study, we assessed time trends in use of sperm cryopreservation by men with TC from 1990 to 2013 in France.

**Methods:**

We collected data from patients diagnosed with TC who underwent sperm cryopreservation in the French national network of sperm banks. Trends in the incidence of sperm cryopreservation were estimated through two statistical models: the commonly used Poisson regression model and the Verhulst model.

**Results:**

Between 1990 and 2013, the overall incidence of sperm cryopreservation rose from 1.73 to 5.57 per 100,000 person-years. Poisson regression predicted an incidence of 9 per 100,000 [95% CI = 8.66–9.34] in 2020. However, since 2005, the observed sperm cryopreservation rate seems to be attenuating. The Verhulst model predicted an incidence of 6 per 100,000 after 2020.

**Conclusions:**

Limitations include the impossibility of analyzing age-standardized incidence. Based on the Verhulst model, results suggest that it is still relevant to follow up TC incidence and sperm cryopreservation in order to confirm or refute the potential decrease already observed in this disease.

## Resume

**Contexte:**

Le cancer des testicules (CT) représente environ 1% de l’ensemble des cas de cancer chez les hommes, mais il demeure néanmoins le cancer le plus fréquent chez les adolescents et les jeunes adultes dans les pays industrialisés. Dans cette étude, nous avons évalué les variations temporelles des autoconservations de sperme réalisées par les hommes atteints d’un CT entre 1990 et 2013 en France.

**Méthodes:**

Les données proviennent des autoconservations de sperme réalisées auprès de patients diagnostiqués avec CT, issues du réseau national français des banques de sperme. Les tendances de l’incidence des autoconservations de sperme ont été estimées à l’aide de deux modèles statistiques: la régression de Poisson, couramment utilisée, et le modèle de Verhulst.

**Résultats:**

Entre 1990 et 2013, l’incidence globale des autoconservations de sperme est. passée de 1,73 à 5,57 pour 100,000 personnes-années. La régression de Poisson montre une estimation de l’incidence de près de 9 pour 100,000 [IC à 95% = 8,66-9,34] en 2020. Cependant, depuis 2005, le taux observé des autoconservations de sperme semble s’atténuer. Le modèle de Verhulst estime alors une incidence aux alentours de 6 pour 100,000 après 2020.

**Conclusions:**

Les limites de cette étude comprennent l’impossibilité d’analyser l’incidence standardisée sur l’âge. Sur la base du modèle de Verhulst, les résultats suggèrent qu’il est. toujours et encore pertinent d’étudier l’évolution de l’incidence du cancer du testicule et des autoconservations de sperme afin de confirmer ou non la diminution/stagnation potentielle déjà observée dans cette maladie.

## Background

Over the past quarter century and particularly in the last decade, the incidence of testicular cancer (TC) has been rising rapidly while at the same time major advances in therapeutic management have led to improved prognoses and survival rates [1–4]. Although it is a relatively rare disease which accounts for approximately 1% of all new male cancer cases, TC remains the most frequent cancer in adolescents and young adults in industrialized countries [5–8].

An important issue for these young men is how TC and its treatment will affect, transiently or permanently, their future fertility [9, 10]. So far, banking samples of semen before treatment is considered as the most appropriate approach [11]. In France, a unique public network of sperm banks (the Centre d’Etude et de Conservation des Oeufs et du Sperme humain, CECOS) was established in 1973 and covers the whole country through 24 affiliated regional sperm banks. We recently published the trends for 1973–2007 in sperm cryopreservation for TC throughout this French network, showing an increasing use of sperm banking during this period [12].

Assessment of TC incidence rate is a worldwide key public health objective in order to predict future trends towards increase or stabilization of this disease, the rate of incidence increase according to country, and regional trends. Several important facts must be highlighted. Firstly, the highest TC incidence rates are observed in North European countries: Norway (12.7 per 100,000 person-years), Denmark (13.4 per 100,000), but rates are also high in East European countries (9.4 per 100,000 in Slovenia) [13]. Secondly, the steepest increases of incidence are often observed in countries with a formerly lower incidence (especially southern European countries: 5.5% per year in Spain), and affecting countries with the highest TC incidence rates (for example Switzerland with an annual increase of 1.8%, and an incidence rate of 12.7 per 100,000) [13]. Thirdly, during the last decade the rise in incidence rates has stabilized in the United Kingdom, Denmark and Austria [13].

In France, the TC incidence rate was 5.7 per 100,000 in 2000–2004 [8]. However, estimations of testicular cancer incidence were based on specific cancer registries in some regions (six registries). No countrywide cancer register exists in France. Thus, registration of annual TC sperm bank procedures, especially in countries such as France which have a single standardized sperm bank network, provides an opportunity to assess indirectly global TC incidence, and consequently to formulate hypotheses regarding TC incidence trends.

The aim of our study was to estimate testicular cancer incidence trends from time trends in use of sperm cryopreservation over the period 1990–2013 by men with testicular cancer.

## Methods

### Data

In this study, we used data from patients who were diagnosed with testicular germ cell tumors and referred to the CECOS national network of sperm banks for sperm cryopreservation. Information was obtained from the national network of sperm banks (in Alsace, Aquitaine, Auvergne, Basse-Normandie, Bretagne, Centre, Champagne-Ardenne, Franche-Comté, Haute-Normandie, Ile de France, Languedoc-Roussillon, Lorraine, Midi-Pyrénées, Nord-Pas de Calais, Pays de la Loire, Picardie, Provence-Alpes-Côte d’Azur, and Rhône-Alpes) for the period 1990 to 2013.

Incidence was calculated from the number of sperm cryopreservations divided by the total French male population (men aged 18–49 years, with a mean annual number of 17,823,090 person-years, provided by the National Institute of Statistics (INSEE)). Incidence was expressed per 100,000 person-years. The National Cancer Agency (Institut National du Cancer, INCa) provided the estimated incidence of men presenting with testicular cancer between 1990 and 2012 [14].

### Statistical analysis

In order to estimate trends in the incidence of sperm cryopreservation, we used two statistical models.

Firstly, the Poisson regression model, which is commonly used in this type of estimation [15, 16]. The mathematical equation for the Poisson regression model is (*Y*| *t*) = *e*^*log*(exposure) + β ’ t^. Exposure represents the person-years and was calculated on the total French male population. β’ is the estimated coefficient which gives the average annual percent change of incidence of sperm cryopreservation.

Secondly, we used the Verhulst (or growth logistic) model [17, 18] defined through the following mathematical eq. $\mathrm{Y}\left(\mathrm{t}\right)=\frac{Y(0)K}{Y(0)+\left(K-Y(0)\right){e}^{- rt}}$ where Y(0) is the sperm cryopreservation incidence in 1990, and K and r the estimated coefficients. The coefficient r is called the proportionality coefficient or Malthusian parameter (rate of maximum population growth). The second parameter K is called the carrying capacity and gives the maximum sperm cryopreservation incidence or the sperm cryopreservation plateau (level-off). In order to estimate r and K, the mean square error method (MSE) was used. A range of r and K values were first defined and the MSE was then calculated for each r and K value. We selected the couple (r, K) which had the smallest MSE.

Finally, the predictions of trends in sperm cryopreservation incidence given by the two models (the Poisson regression model and the Verhulst model) were compared with the true sperm cryopreservation incidence rate observed in the CECOS network of French regional sperm banks.

The statistical analyses were performed using R software (CRAN) with a significance level of .05.

The datasets used and analyzed during the current study are available from the corresponding author on reasonable request.

## Results

The number of men using sperm cryopreservation rose from 299 in 1990 to 1025 in 2005, a mean increase of 16% per year. The annual increase fell to 1.54% up to 2011, when 1120 men used sperm cryopreservation (Table 1). In 2013, this number decreased to 1012.

**Table 1:** Rates and numbers of men using sperm cryopreservation for testicular cancer in the CECOS network from 1990 to 2013 and predictions after 2013 derived from the Poisson regression model and the Verhulst model

Mean annual number of 100,000 person-years^a^		Rates (number)		
		Men using sperm cryopreservation	Poisson regression model	Verhulst model
1990	172.69	1.73 (299)	2.64 (456)	1.96 (340)
1995	174.33	2.95 (515)	3.24 (565)	3.27 (576)
2000	177.58	4.64 (824)	3.98 (706)	4.48 (802)
2005	183.52	5.59 (1025)	4.88 (895)	5.27 (956)
2010	182.91	5.52 (1009)	5.98 (1094)	5.68 (1037)
2015	183.00	–	7.34 (1343)	5.87 (1073)
2020	183.48	–	9.00 (1652)	5.94 (1089)

^a^Mean annual number of 100,000 person-years means that, for example, in 1990, 17,269,000 men aged between 18 and 49 years old were at risk of developing testicular cancer in France. The incidence of sperm cryopreservation was calculated as the number of sperm cryopreservations divided by the mean annual number of 100,000 person-years for each year

**Fig. 1:**
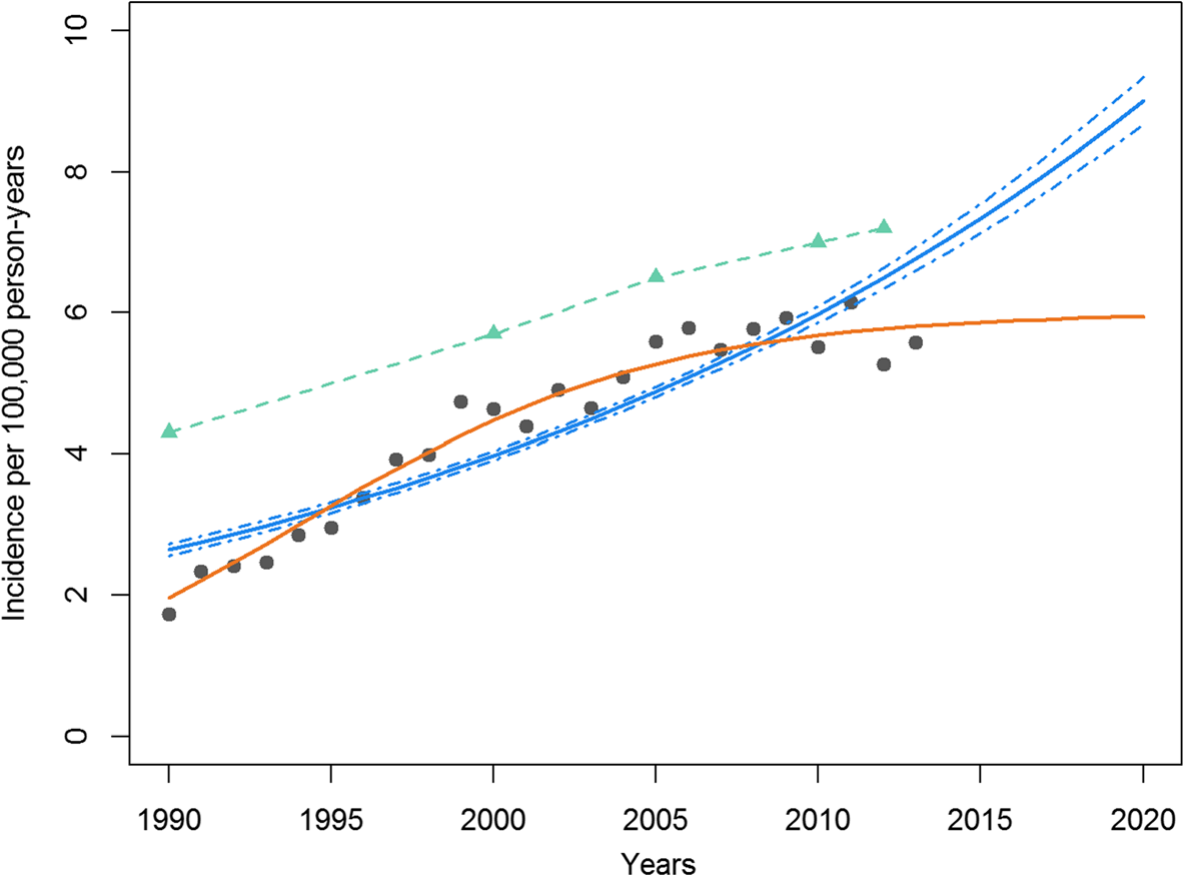
Incidence of sperm cryopreservation for testicular cancer from CECOS network data (France). Estimations of sperm cryopreservation for testicular cancer derived from the Poisson regression model and the Verhulst model from 1990 to 2020, and testicular cancer incidence from National Cancer Institute (INCa) data.  (green triangle) Incidence of testicular cancer in France between 1990 and 2012 taken from [14].  (orange line) Estimated incidence of sperm cryopreservation using the Verhulst model.  (blue line/dashed lines) Estimated incidence of sperm cryopreservation with confidence intervals using the Poisson regression model. (gray point) Observed incidence of sperm cryopreservation provided by CECOS data

In the Fig. 1, two models were performed using the incidence of men with TC who used sperm cryopreservation (gray point) between 1990 and 2013: the Poisson regression model (blue line with confidence intervals) and the Verhulst model (single orange line). The estimated incidence of men with TC in France (INCa data) is shown in green (dashed line with triangle).

The overall incidence of sperm cryopreservation rose from 1.73 to 5.57 per 100,000 between 1990 and 2013. The maximum incidence was observed in 2011 with 6.14 per 100,000.

The Poisson regression model predicted an incidence rising from 2.64 to 6.76 per 100,000 between 1990 and 2013, and suggested that the incidence would reach 9.00 per 100,000 [95% confidence interval CI = 8.66–9.34] in 2020. The annual percent change was estimated at 4.16% [95% CI = 3.98–4.39]. The Poisson regression model suggested that the incidence would increase after 2013. However, the Figure shows that the predictions performed by this model do not seem to fit the trend and were above the observed incidence of sperm cryopreservation. After 2005, the data are in favor of a pause in the increase of sperm cryopreservation incidence.

The Verhulst model was more in line with the observed data, showing attenuation in the incidence of sperm cryopreservation after 2005. Predictions performed by this model showed an incidence of sperm cryopreservation rising from 1.96 to 5.81 per 100,000 between 1990 and 2013, and to 5.98 per 100,000 in 2020. The Verhulst model estimated that the maximum incidence of sperm cryopreservation will be 6 per 100,000 after 2020, which will represent 1100 men visiting a sperm bank.

The trends estimated by the Verhulst model are much closer to the estimated TC incidence based on the National Cancer Institute data than the Poisson regression trends.

## Discussion

Our results showed strong evidence for increasing use of sperm cryopreservation by men with TC between 1990 and 2013, rising from 1.73 to 5.57 per 100,000 person-years. Interestingly, this annual increase is in agreement with the TC incidence observed in other studies worldwide [8, 13, 19].

However, we observed a slight attenuation beginning after 2005, as the incidence decreased from 5.58 in 2005 to 5.57 in 2013. A similar decrease in the incidence rate of TC has recently been reported in other countries in Northern Europe, in the United States and Australia [8, 20]. Although this study does not show attenuation in France, the testicular cancer data were only collected up to 2004. The same remark could be made regarding the study of Le Cornet et al. [19]. They showed that the greatest increase of TC incidence was in France, with over 2500 new cases estimated in 2025. However, this estimation was calculated for the period 1988–2007. In the light of these findings, our observed trends and attenuation need to be strengthened by prospective studies on TC.

In order to explain this transition, Znaor et al. studied generational transitions in 38 countries and showed that a deceleration in risk occurred in younger generations born in the 1960s and 1970s, but only in the most developed countries [20]. This generational effect had already been observed for men born during World War II. It has been suggested that modifications in lifestyle exposure to etiological factors for TC could account for these two changes.

In our study, we used two methods to model the number of sperm cryopreservations for TC between 1990 and 2013. Poisson regression is a commonly used method for modeling count data in cancers such as TC (rare events with few new cases per year [16]). However, in this method, the estimated coefficient is a constant rate parameter which cannot take into account changes in data trend. Sperm cryopreservation observations throughout the CECOS network showed a higher increase before 2005 and an attenuation afterwards. The second method, the Verhulst model, assumes that the growth rate (or incidence) will tend to the value K, which is here the maximum sperm cryopreservation incidence. This model could therefore take into account trend changes in incidence and therefore the attenuation in sperm cryopreservation before TC that was recorded by the CECOS sperm bank network.

### Limitations

Although the crude sperm cryopreservation incidence observed in our series was not age-standardized (our data were totally anonymous), it is still a good indirect indicator of the trend of the true incidence of TC. From 1994 onwards, sperm cryopreservation was systematically performed before cancer treatment and the samples were stored in the French regional sperm bank network (CECOS). Within recent decades, pediatricians and uro-andrologists have made every effort to provide adolescents and young adults diagnosed with TC with the possibility of sperm banking in order to preserve their ability to procreate. Although not all patients with TC wish to perform sperm cryopreservation, we have observed an increasing number of these procedures over the last 20 years, leading to a good correlation between the total number of TC and the number of sperm cryopreservation procedures.

## Conclusions

Sperm cryopreservation incidence in TC confirms a trend toward a slight attenuation or plateau in the TC incidence rate. With regard to TC incidence, the Verhulst model seems more efficient than the Poisson model. As a matter of public health policy, it is important to follow up TC incidence and sperm cryopreservation so that the decrease in this disease can be confirmed or refuted. In the meantime, it remains and will always remain important to identify the causes of TC and trends in the incidence of the disease, and to protect the health and future fertility of young men.

## Abbreviations

CECOS: Centre d’Etude et de Conservation des Oeufs et du Sperme humain
TC: testicular cancer
